# Succinate Anaplerosis Has an Onco-Driving Potential in Prostate Cancer Cells

**DOI:** 10.3390/cancers13071727

**Published:** 2021-04-06

**Authors:** Ana Carolina B. Sant’Anna-Silva, Juan A. Perez-Valencia, Marco Sciacovelli, Claude Lalou, Saharnaz Sarlak, Laura Tronci, Efterpi Nikitopoulou, Andras T. Meszaros, Christian Frezza, Rodrigue Rossignol, Erich Gnaiger, Helmut Klocker

**Affiliations:** 1 Daniel Swarovski Research Laboratory, Department of Visceral, Transplant and Thoracic Surgery, Medical University Innsbruck, 6020 Innsbruck, Austria; andras.meszaros@i-med.ac.at (A.T.M.); erich.gnaiger@oroboros.at (E.G.); 2 Oroboros Instruments GmbH, 6020 Innsbruck, Austria; 3 Institute of Human Genetics, Medical University Innsbruck, 6020 Innsbruck, Austria; juan.perez-valencia@uibk.ac.at; 4 Medical Research Council Cancer Unit, University of Cambridge, Cambridge CB2 0XZ, UK; ms2122@mrc-cu.cam.ac.uk (M.S.); laura.tronci@external.ifom.eu (L.T.); en338@mrc-cu.cam.ac.uk (E.N.); cf366@mrc-cu.cam.ac.uk (C.F.); 5 Institut National de la Santé Et de la Recherche Médicale (INSERM) U1211, Bordeaux University, 33076 Bordeaux, France; claude.lalou@u-bordeaux.fr (C.L.); saharnaz.sarlak@u-bordeaux.fr (S.S.); rodrigue.rossignol@u-bordeaux.fr (R.R.); 6 Department of Surgery, Division of Experimental Urology, University Hospital for Urology, Medical University Innsbruck, 6020 Innsbruck, Austria

**Keywords:** cancer metabolism, prostate cancer, anaplerosis, mitochondria, succinate

## Abstract

**Simple Summary:**

Depending on the availability of nutrients and increased metabolic demands, tumor cells rearrange their metabolism to survive and, ultimately, proliferate. Here, the authors investigated the effect of succinate, a metabolite of the mitochondrial citric acid cycle, on malignant and non-malignant prostate cells. They analyzed uptake through membrane transporters and intracellular accumulation, which subsequently fuels metabolism and enhances oncogenic properties of the tumor cells. The findings shed light to the metabolic adaptations that prostate tumor cells undergo, providing a better understanding of metabolic rewiring and strategies for therapeutic intervention.

**Abstract:**

Tumor cells display metabolic alterations when compared to non-transformed cells. These characteristics are crucial for tumor development, maintenance and survival providing energy supplies and molecular precursors. Anaplerosis is the property of replenishing the TCA cycle, the hub of carbon metabolism, participating in the biosynthesis of precursors for building blocks or signaling molecules. In advanced prostate cancer, an upshift of succinate-driven oxidative phosphorylation via mitochondrial Complex II was reported. Here, using untargeted metabolomics, we found succinate accumulation mainly in malignant cells and an anaplerotic effect contributing to biosynthesis, amino acid, and carbon metabolism. Succinate also stimulated oxygen consumption. Malignant prostate cells displayed higher mitochondrial affinity for succinate when compared to non-malignant prostate cells and the succinate-driven accumulation of metabolites induced expression of mitochondrial complex subunits and their activities. Moreover, extracellular succinate stimulated migration, invasion, and colony formation. Several enzymes linked to accumulated metabolites in the malignant cells were found upregulated in tumor tissue datasets, particularly NME1 and SHMT2 mRNA expression. High expression of the two genes was associated with shorter disease-free survival in prostate cancer cohorts. Moreover, *in-vitro* expression of both genes was enhanced in prostate cancer cells upon succinate stimulation. In conclusion, the data indicate that uptake of succinate from the tumor environment has an anaplerotic effect that enhances the malignant potential of prostate cancer cells.

## 1. Introduction

During transformation, cells reprogram metabolism altering biosynthetic and bioenergetic pathways to guarantee biomass for proliferation, redox control and energy turnover [1,2].

In 1924, Otto Warburg described the first and most studied metabolic alteration in malignant cells namely aerobic glycolysis [3,4], in which cancer cells import glucose and export lactate even in the presence of oxygen, supposedly due to dysfunction in oxidative metabolism (i.e., mitochondrial respiration). However, many studies have shown that mitochondrial function is essential for tumor growth [5], and changes in expression of glucose transporters and glycolytic enzymes are more associated with proliferating cells rather than solely malignant transformation [1]. For instance, the tricarboxylic acid (TCA) cycle generates intermediates that support biosynthesis of lipids, amino acids, and nucleotides. These metabolites are necessary for other metabolic pathways, as well as proliferation [6].

Succinate is a dicarboxylic acid metabolized to fumarate through an enzymatic complex termed succinate dehydrogenase (SDH). As the largest component of the mt-inner membrane Complex II (CII), SDH is constituted by four subunits, in which SDHA and SDHB function as the catalytic domains in the mitochondrial matrix, attached to SDHC and SDHD in the mt-inner membrane [7]. SDH couples the oxidation of succinate and FAD to fumarate and FADH_2_ in the TCA cycle with electron transfer (ET) to ubiquinone. Therefore, dysregulation in SDH may exert effects on many metabolic pathways [8] and bioenergetics.

Even though it was reported that some cancer cells can harbor mutations in all SDH subunits, the most commonly found is a loss of function mutation in the Fe-S cluster of the catalytic subunit SDHB, which is highly associated to malignancy [9]. The inhibition of SDH leads to an impairment of the TCA cycle and respiration, resulting in accumulation of succinate [8,10,11]. This accumulation leads to transmission of an “oncogenic” signal from mitochondria to the cytosol, where the hypoxia inducible factor 1α (HIF-1α) prolyl hydroxylases (PHDs) are inhibited [12]. This process leads to HIF-1α stabilization under normoxic conditions [13] and elicits a “pseudohypoxic” state responsible for increased expression of genes involved in adaptation to hypoxia, diminished oxidative phosphorylation (OXPHOS) and stimulated glycolysis [10], even in the presence of oxygen.

Succinate also plays a role in other cellular processes. For instance, succinate is released in the microenvironment, e.g., by macrophages, suppressing neuroinflammation via a succinate-SUCNR1-dependent pathways [14]. In heart muscle, accumulation of succinate may result in cardiac ischemia-reperfusion injury due to mitochondrial ROS generated through reverse electron transfer (RET) [15,16]. Moreover, succinate is found increased in body fluids [17] in some pathological conditions [18], such as diabetes [19,20], obesity [20], and hypertension [21]. Its presence in the extracellular matrix can be used to monitor and diagnose colorectal cancer [22] and hepatocellular carcinoma [23].

In prostate cancer, combined metabolomics and transcriptomics analysis of tissue samples revealed increased levels of TCA metabolites and upregulated expression of related enzyme genes, which were correlated with tumor grade [24]. Our recent study on OXPHOS capacities in prostate cancer tissues uncovered increased oxidation of succinate via CII as a compensation for a decreased capacity to oxidize substrates via Complex I (CI) in high-grade prostate tumors [25].

The succinate supply in prostate tumor cells may originate from cellular metabolism or by uptake from external sources. Succinate may be imported from the external microenvironment across the plasma membrane [26] via sodium-dependent dicarboxylate transporters, fueling succinate-driven OXPHOS in mitochondria. However, it is still unknown how critical is the uptake of succinate for tumorigenesis and malignant cell metabolism.

In this study, the fate of exogenous succinate in prostate cancer cells was investigated towards energy transformation, mitochondrial physiology, and tumorigenic potential. Our findings suggest that succinate enhances the tumor potential and support exploration of metabolic targets for new strategies for prostate cancer treatment.

## 2. Materials and Methods

### 2.1. Cell Lines and Culturing Conditions

Human prostate cell lines were purchased from the American Type Culture Collection (ATCC; Rockville, MD, USA) and maintained at 37  °C in humidified atmosphere at 5% CO_2_. RWPE-1 cells (immortalized cells from non-tumorigenic prostate tissue) were cultured in keratinocyte serum free medium (K-SFM, Cat. N. 17005034, Gibco^®^, Thermo Fisher Scientific; Waltham, MA, USA) supplemented with 0.05 mg/mL bovine pituitary extract and 5 ng/mL human recombinant epidermal growth factor. LNCaP cells (malignant prostate cells derived from a lymph node metastasis) were cultured in DMEM 1.0 g/L glucose (Cat. N. P04-01550, Pan Biotech, Aidenbach, Germany,) supplemented with 10% fetal bovine serum (Cat. N. 10270106, Gibco^®^), 1% HEPES 1M pH 7.2 and 1% penicillin-streptomycin (Gibco^®^). All cells were checked periodically for mycoplasma infection and tested non-infected.

### 2.2. Protein Extraction and Immunoblotting

Cells were seeded in 6-well plates in approximately 1.0 × 10^6^ cells/well. When 70–80% confluent, fresh medium was added and medium was supplemented with either sodium succinate dibasic hexa-hydrate (Sigma-Aldrich; St Louis, MO, USA) to a final concentration of 5 mM or vehicle alone (water, control). After a 6 h incubation, cells were washed with PBS and 200 μL of 1× RIPA buffer (Abcam; Cambridge, UK) with protease and phosphatase inhibitors (Thermo Fisher ) was added in each well. Cells were scraped-off, collected and frozen at −80 °C until further use.

For protein extraction and immunoblotting, cell lysates were thawed, sonicated and NuPAGE™ LDS Sample Buffer (Thermo Fisher) was added and sample volume was adjusted to equal protein concentration. Samples were heated at 90 °C for 5 min and 15–20 μg of protein extract per lane was loaded on a 10 % SDS-PAGE gel. After running at 100 V for 1.5 h, the SDS-PAGE gels were transferred to a blotting membrane using iBlot 2 Dry Blotting System IB23002 (Invitrogen; Carlsbad, CA, USA). The membranes were then placed in REVERT total protein stain solution (LI-COR Biosciences; Lincoln, NE, USA), washed and scanned using an Odyssey CLx Imaging System (LI-COR) for acquisition of total protein staining. Subsequently, the membranes were blocked with 5% non-fat milk in TBS-Tween 1% for 1 h and then incubated with primary antibodies diluted in 5% non-fat milk + TBS-Tween 1% or TBS-Tween 1% + 0.02% azide overnight at 4 °C.

The following primary antibodies were used for immunoblotting: anti-SLC13A5 (1:400 Thermo Fisher), anti-SLC13A3 (1:1,000 Aviva Systems Biology; San Diego, CA, USA), anti-β-actin (1:10,000 Sigma-Aldrich), anti-SDHA (1:1,000 Abcam); the total OXPHOS Rodent WB Antibody Cocktail (1:1,000 Abcam) containing the antibodies anti-NDUFB8, anti-SDHB, anti-UQCRC2, anti-COX II and anti-ATP5 as a premixed solution. After 3 washes in TBS-Tween 1%, membranes were incubated for 1 h in the dark at RT with the appropriate HRP-conjugated secondary antibodies: anti-rabbit HRP conjugated secondary (1:10,000 Thermo Fisher) or anti-mouse HRP conjugated secondary (1:10,000 Thermo Fisher). The protein acquisitions and analyses were done using Image Studio™ Software (LI-COR) and normalized by total protein (see Supplementary Materials). All western blot raw images are provided in Supplementary Materials, in Figure S6.

### 2.3. Metabolite Extraction and Liquid Chromatography-Mass Spectrometry (LC-MS) for Metabolomics Analysis

Cell lines were incubated for 1, 3, 6, 12 or 24 h in their specific cell culture media supplemented with 5 mM succinate disodium salt or vehicle. For isotope tracing, cells were incubated for 6 h with or without 5 mM succinic acid-^13^C_4_ (99% Sigma-Aldrich). Cells were then washed three times with PBS and extraction buffer (50% LC-MS grade methanol and 30% acetonitrile, 20% ultrapure water) was added in a ratio of 1 mL/10^6^ cells. Following 15 min incubation in dry ice, the cells were scraped off, kept under vigorous shaking for 15 min at 4 °C and incubated at −20 °C for 1 h. After centrifugation, the samples were stored in autosampler glass vials at −80 °C until further analysis. 

Prior to the run, samples were randomized to avoid bias due to machine drift and processed blindly. To perform the metabolomics analysis, a Q Exactive Orbitrap mass spectrometer (Thermo Fisher) coupled to a Dionex U3000 UHPLC (Thermo Fisher) was utilized. For the chromatographic separation, a ZIC-HILIC column (150 × 4.6 mm^2^) and a ZIC-pHILIC column (150 mm × 2.1 mm^2^) with respective guard columns (20 × 2.1 mm^2^, 5 µm) (all Merck Millipore; Burlington, MA, USA) were used. 

For the ZIC-pHILIC column, the temperature was set at 40 °C, the flow was at 200 μL/min and the mobile phase was comprised of 20 mM ammonium carbonate and 0.05% ammonium hydroxide in water with pH 9.4 (solvent A), and acetonitrile (solvent B). The chromatographic gradient was run as follows (time, % solvent B): 0−2 min, 80%; 2−17 min, linear gradient from 80% to 20%; 17−17.1 min, linear gradient from 20% to 80%; 17.1−22.5 min, hold at 80%.

For the ZIC-HILIC column the flow rate was set at 300 μL/min while the column oven temperature was set at 35 °C, and the gradient comprised of 0.1% formic acid in water with pH 2.7 (solvent A) and 0.1% formic acid in acetonitrile (solvent B). The gradient run as follows (time, % solvent B): 0, 80%; 12 min, 50%; 26 min, 50%; 28 min, 20%; 36 min, 20 %; 37 min, 80%; 45 min, 80%.

Q Exactive was operating at full MS and polarity switching mode. Standards for the ^13^C succinate (CK Isotopes; Leicester, UK) and succinate (Sigma-Aldrich) were utilized to verify the retention times of these compounds and assist the identification. X Calibur 4.1 software (Thermo Fisher) was used to perform the peak integrations and spectra analysis by reference to an internal library of compounds.

### 2.4. Enzyme Activities

For enzyme activities, 1.0 × 10^7^ cells were harvested using trypsin-EDTA 0.25%, washed with phosphate saline buffer, and a cell pellet was obtained by centrifugation at 300 *g* for 5 min and snapped frozen at −80 °C. After collection of all samples, the cell pellets were thawed on ice, suspended in protein extraction buffer containing 20 mM Tris-HCl pH 7.2, 250 mM saccarose, 40 mM KCl, 2 mM EGTA, 1 mg/mL BSA and mechanical cell lysis was performed using a glass potter at 4 °C. After centrifugation at 500 *g* for 20 min, collection of supernatant, and another centrifugation at 650 g for 20 min, cell homogenates were obtained, and protein concentration was determined using the BCA assay (Thermo Fisher). The activity of ETS Complexes I, II, III and citrate synthase (CS) were measured using standardized protocols, as described previously [27]. The assays were carried out spectrophotometrically at 30 °C, using a double wavelength Xenius spectrophotometer (SAFAS; Monaco, France). Activities were measured using 40 µg total protein per sample and expressed in nmol*min^−1^*mg^−1^ protein. CIV activity was measured using the Oroboros O2k [28] (Oroboros Instruments; Innsbruck, Austria) after inhibition of CIII by antimycin A, addition of tetramethyl-*p*-phenylenediamine (TMPD) and ascorbate, and final inhibition of CIV using azide. Respiratory rates were corrected for chemical autoxidation and instrumental O_2_ background and normalized per cell count; O_2_ flow per cell is expressed in units [amol*s^−1^*x^−1^], where “x” represents the elementary unit of cell count [29].

### 2.5. Dehydrogenase Activity

Cell dehydrogenase activity was determined using a WST-1 assay (Roche; Basel, Switzerland), which is based on cleaving the tetrazolium salt WST-1 to formazan. Briefly, cells were incubated with the WST-1 reagent (1:10) for 1 h and then absorbance was measured on a Chameleon 5025 (HVD Life Sciences; Vienna, Austria) at 490 nm.

### 2.6. High-Resolution Respirometry

Cell viability was measured before each experiment with trypan blue staining (Sigma-Aldrich) using a Countess II FL Automated Cell Counter (Thermo Fisher). Respiratory measurements were performed with samples exhibiting 90% or higher cell viability. High-resolution respirometry (HRR) using several O2k (Oroboros Instruments) in parallel was performed to determine oxygen concentration and oxygen consumption with application of substrate-uncoupler-inhibitor titration (SUIT) protocols at 37 °C under 750 rpm stirrer speed [28]. DatLab 7 software (Oroboros Instruments) was used for real-time data acquisition at a recording interval of 2 s and for data analysis. For HRR analysis in living cells, 1.0 × 10^6^ x/mL of RWPE-1 or LNCaP cells suspended in the respective basal cell culture media without supplements (fetal calf serum and bovine pituitary extract, respectively) and were placed into the pre-calibrated 2 mL O2k-chamber (more information on www.bioblast.at (accessed on 1 February 2020)). Mitochondrial respiration medium (Oroboros MiR05-Kit) containing 110 mM sucrose, 60 mM K^+^-lactobionate, 0.5 mM EGTA, 3 mM MgCl_2_, 20 mM taurine, 10 mM KH_2_PO_4_, 20 mM HEPES, pH 7.1 [30] was used for respirometric measurements with permeabilized cells. Respiratory capacities were expressed as O_2_ flow (*I*_O2_) [amol*s^−1^*x^−1^], where “x” represents the elementary unit of cell count [29] and corrected for residual oxygen consumption (*Rox*) determined in each SUIT protocol. For determination of flux control ratios (*FCR*), respiratory capacities were normalized to an internal reference rate within each experiment. 

In permeabilized cells, reference protocol SUIT-001 O2 ce-pce D003 was performed to evaluate respiratory control in several pathway control states and such coupling states (LEAK, OXPHOS, and ET), as previously described [28,29]. 

Rotenone, adenosine 5’diphosphate potassium salt, cytochrome *c*, succinate disodium salt, carbonyl cyanide m-chlorophenyl hydrazone, antimycin A and digitonin were purchased from Sigma-Aldrich. Details on preparation of solutions and calculations for flux control efficiencies [31] are available at www.bioblast.at (accessed on 1 February 2020).

### 2.7. ATP Content

The steady-state intracellular ATP content was measured using the CellTiter-Glo^®^ Luminescent Cell Viability Assay (Promega; Madison, WI, USA) following manufacturer’s instructions. Luminescence was monitored on a Clariostar plate reader (Promega) and values were calculated based on an ATP standard curve. Data were normalized to cell count.

### 2.8. Cell Proliferation 

Cells were incubated at 37 °C with 0.5 mM or 5 mM of succinate or vehicle for a period of 120 h in 24-well plates. Light microscopy images were acquired using an IncuCyte^®^ instrument (Sartorius AG; Goettingen, Germany) with record intervals of 2 h. Cell proliferation was quantified by the instrument software, counting the number of phase objects over time. After setting up the morphology of individual cells, a label-free count was done until the point that cultures became densely packed and cell edges could not be accurately determined.

### 2.9. Transwell Migration and Invasion

Migration and invasion were determined by a two-chamber transwell assay (Corning Incorporated; Corning, NY, USA). The upper side of the polycarbonate film was left untreated (migration) prior to cell seeding or was covered with Matrigel™ (500 ng/μL; BD Biosciences; Franklin Lakes, NJ, USA) (invasion, chemotaxis assay). 600 μL of complete medium was added into the lower chamber. After succinate addition, 2 × 10^4^ cells were suspended in 100 μL supplement-free medium and added into the upper chamber (inserts). After incubation for 24 h at 37  °C, transwells were gently picked up, invaded cells on the bottom of the inserts were rinsed with PBS. Nucleic acids were stained with 0.05% crystal violet and photographed with a binocular loop (Leica; Wetzlar, Germany) microscope equipped with a Mrc camera (Zeiss; Oberkochen, Germany). Cells were counted using the ImageJ software (NIH; Bethesda, MD, USA).

### 2.10. Anchorage-Independent Growth in Soft Agar

Six-well plates were coated with 1 mL of 1.6% low melting agarose (Sigma-Aldrich) in cell culture medium and stored at 4 °C for at least 30 min to let the agarose solidify. Afterwards, 1 × 10^5^ cells/well were suspended in 1 mL of 0.8% low melting agarose medium mixture and seeded. Plates were then incubated at 4 °C for 15 min to allow solidifying the upper layer of the gel. Complete cell culture medium was added on top of the wells and plates were incubated at 37 °C in humidified atmosphere of 5% CO_2_ for 2–3 weeks. Number of colonies were counted using ImageJ software (NIH).

### 2.11. RNA Extraction, cDNA Synthesis and Quantitative Real Time PCR (qRT-PCR) 

Cells were incubated at 37 °C with 5 mM of succinate or vehicle for 48 h. Total RNAs were extracted from cells using EXTRACTME total RNA kit (Blirt S.A.; Gdańsk, Poland) according to manufacturer’s recommendations, along with an additional purification step by DNase treatment to guarantee elimination of any genomic DNA. The integrity and quantity of total RNA was determined using a NanoDrop 1000 spectrophotometer (Thermo Fisher). For all cases, 1 µg RNA was used for cDNA synthesis using LunaScript™ RT SuperMix Kit (New England Biolabs; Ipswich, MA, USA). Quantitative RT-PCRs were performed using the following predesigned TaqMan Gene Expression Assays (Thermo Fisher): NME1/NME1-NME2 (Hs00264824_m1), SHMT2 (Hs01059263_g1) and HPRT1 (Hs02800695_m1). Results were analyzed using the comparative Ct method, in which each sample was normalized to its internal reference, mRNA levels of the HPRT1 gene, to account for any potential cDNA loading differences. Acquisition and analysis were done using the CFX Connect Real-Time PCR Detection System (Bio-Rad; Hercules, CA, USA).

### 2.12. In silico Analysis of Prostate Cancer Gene Expression Data

In silico analysis was performed using the Cancer Tool [32] (http://web.bioinformatics.cicbiogune.es/CANCERTOOL/index.html. Accessed on 20.01.2021). Genes of interest were searched in several publicly available prostate cancer transcriptome datasets that are incorporated to the Cancer Tool and violin plots were generated. Analyzes show the comparison between the non-malignant (N) and primary tumor (PT) tissue samples; and the N, PT and metastatic (M) samples. Cancer Tool also generated disease-free survival (DFS) analysis Kaplan-Meier data divided into 4 quartiles according to gene expression levels. Datasets used: Glinsky et al. [33]; Grasso et al. [34]; Taylor et al. [35]; Varambally et al. [36]; TCGA-PRAD (https://portal.gdc.cancer.gov/projects/TCGA-PRAD. Accessed on 01.02.2021). 

### 2.13. Statistical Analysis

Statistical significance was determined by unpaired Student’s *t*-test comparing two independent groups. Multiple groups comparison was performed using ordinary one-way or two-way Anova with Tukey’s or Mann-Whitney post-corrections. Statistical significance was considered at α  ≥  0.05. All experiments were performed with a minimum of 3 biological replicates, and whenever possible, 2 or more technical replicates. The results were expressed as median and interquartile range (IQR) for *N* independent experiments and *n* technical repeats. Prism 8 (GraphPad Software; San Diego, CA, USA) was used for all statistics calculations.

## 3. Results

### 3.1. Prostate Cells Express Plasma Membrane Dicarboxylic Transporters, Take up and Accumulate Succinate

**Figure 1:**
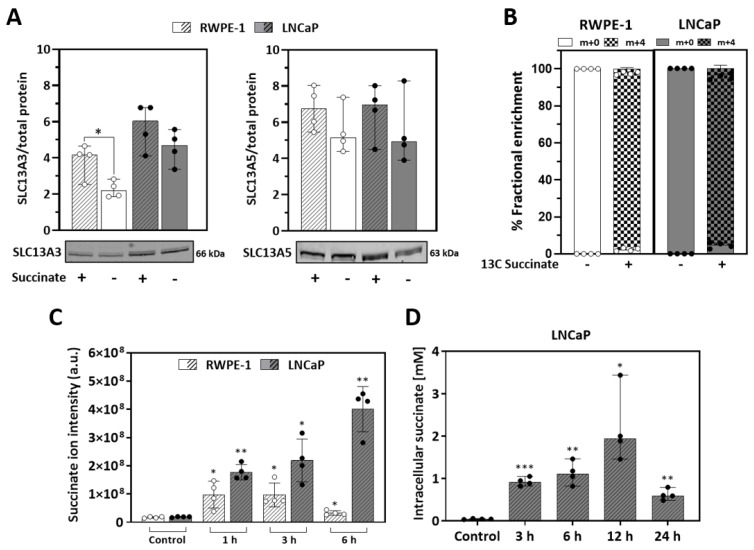
Succinate transport and accumulation. Expression of dicarboxylate transporters and succinate uptake in RWPE-1 and LNCaP cells incubated for 6 h (**A**–**C**) or up to 24 h (**D**) with 5 mM succinate or vehicle as controls. (**A**) SLC13A3 (NaDC3) and SLC13A5 (NaCT) expression. Left, representative western blot image; right, quantification of protein band intensities relative to total protein content within each extract of RWPE-1 and LNCaP cells (*N* = 4). (**B**) Succinate uptake (% of total fractional enrichment) analyzed by target metabolomics. Cells were incubated for 6 h with 5 mM succinic acid-^13^C_4_ or the same volume of vehicle as control (*N* = 1; *n* = 4) (**C**) Transient intracellular succinate accumulation measured by untargeted LC-MS as succinate ion intensity (*N* ≥ 2; *n* = 4). (**D**) Intracellular succinate concentration [mM] in LNCaP cells. (*N* = 1; *n* = 4). Median and IQR. Unpaired *t*-tests comparing +/- succinate in each condition (**A**,**B**) one or two-way ANOVA and Tukey’s multiple comparisons tests (**C**,**D**). * *p* = 0.05; ** *p* < 0.001; *** *p* < 0.0001.

Expression of the high-affinity plasma membrane transporter for succinate, NaDC3 (sodium-dependent dicarboxylate transporter member 3, SLC13A3 gene) and/or the low affinity transporter NaCT (sodium-dependent citrate transporter member 5, SLC13A5 gene), represent key factors for succinate uptake [37,38]. We analyzed transporter protein expression by western blot in non-malignant RWPE-1 and malignant LNCaP prostate cells after propagation in either 5 mM of succinate or vehicle. Beforehand, using high-resolution respirometry, the effect of increasing concentrations of succinate on respiration was evaluated, to choose a standard concentration of 5 mM for the subsequent experiments, which triggered optimal stimulation (Figure S1). The expression of both transporters in non-malignant RWPE-1 and malignant LNCaP cells with or without incubation for 6 h with succinate (5 mM) was confirmed (Figure 1A). The presence of succinate increased SLC13A3 transporter expression in RWPE-1 but not significantly in LNCaP cells.

To evaluate the succinate transport and uptake across the plasma membrane, metabolomic analysis was performed with cells incubated with 5 mM succinic acid-^13^C_4_. After 6 h approximately 99% of all intracellular succinate content was labelled, showing that both cell lines are capable to transport external succinate (Figure 1B). A time course experiment with extracellular non-labelled succinate for 1, 3 or 6 h showed that both cell lines internalize succinate although to a different extent (Figure 1C). LNCaP cells displayed higher uptake rates than RWPE-1, achieving a peak of approximately 2 mM intracellular succinate concentration after 12 h (Figure 1D). These data show that the malignant LNCaP cells are more prone to accumulate succinate than the non-malignant RWPE-1 cells.

### 3.2. Succinate Uptake Induces Anaplerosis and Amino Acid Biosynthesis

**Figure 2:**
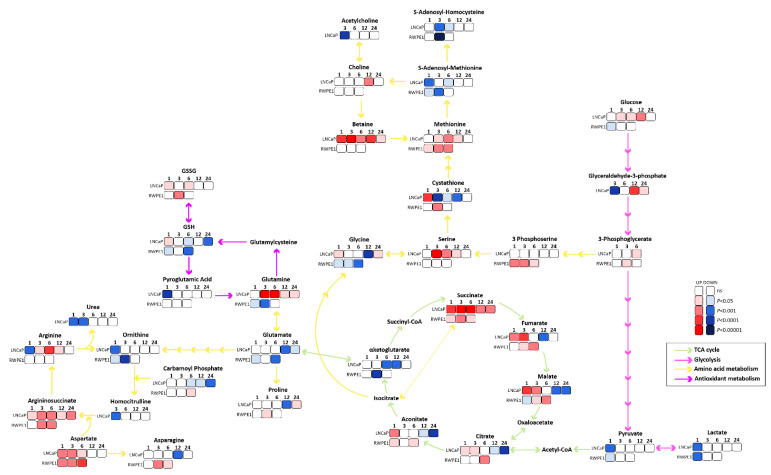
Metabolite alterations upon succinate stimulation. Metabolomics pathway network map of main pathways affected by succinate, TCA cycle (green arrows), glycolysis (pink arrows), amino acids metabolism (yellow arrows) and antioxidant metabolism (purple arrows) pathways are shown. Significantly increased metabolites are in red scale and significantly decreased are in blue scale. The darker the color, the higher the statistical significance, white squares represent no statistical difference to control. (*N* ≥ 4).

After the observation of external succinate accumulation to a different extent in the cell lines, metabolite levels achieved with succinate incubation were determined. Potential pathways that are triggered by succinate accumulation were subsequently identified based on the enzymes linked to changed metabolites (Table S1). The main metabolic changes are depicted in Figure 2. In LNCaP, upregulation in TCA cycle metabolites is observed at time points up to 6 h (Figure 2). Thereafter succinate did not stimulate the production of the other intermediates of the TCA cycle, and instead redirection to other metabolic pathways such as glycolysis and amino acid metabolism was observed. In RWPE-1, succinate fueled primarily into TCA metabolites and some amino acids. The main similarities between both cell lines were accumulation of methionine, argininosuccinate, and aspartate and depletion of S-adenosyl-homocysteine, S-adenosyl-methionine, pyruvate, lactate, αKG, and ornithine. Exclusively in LNCaP cells, there was accumulation of betaine, serine, glucose, glutamine, arginine, and reduction of glutamate, urea, GSH and cystathionine. In RWPE-1 cells, there was accumulation of 3-phosphoserine, fumarate, malate, citrate, aconitate, asparagine, proline and carbamoyl-phosphate and reduction of glycine, glutamate and GSH. In summary, this result suggests that succinate taken up by prostate cells exerts an anaplerotic effect and stimulates amino acid metabolism, particularly of methionine, argininosuccinate, and aspartate.

To highlight the metabolic changes induced by succinate incubation and associate metabolite alterations to biological function, the metabolic pathways affected by succinate were further investigated. Enzymes involved in the production of the altered metabolites were identified (Table S2) providing a list of 148 proteins (Table S3A–C). Using STRING software, the KEGG pathways in which the enzymes are involved in were annotated (Table S4A,B). The number of enzymes associated with INCREASED; REDUCED or UNCHANGED metabolites after 1 or 6 h of succinate stimulation were then summed up for each KEGG pathway and pathways were grouped into seven biological function groups: (i) energy metabolism; (ii) amino acids metabolism; (iii) proliferation; (iv) fatty acid metabolism; (v) motility and cell adhesion; (vi) cell death and, (vii) chronic diseases (Figure S2A,B). Figure S2C displays contributing altered metabolites associated with the biological function groups in RWPE-1 and LNCaP cells, respectively, after 1 and 6 h.

Taken together, the metabolomics analyzes suggest that anaplerotic pathways were mainly affected by succinate addition in both cell lines, although to a different extent, mostly through the biosynthesis and metabolism of amino acids, and carbon metabolism pathways, mainly one carbon metabolism.

### 3.3. Succinate Supports Mitochondrial Activity and Respiration

**Figure 3:**
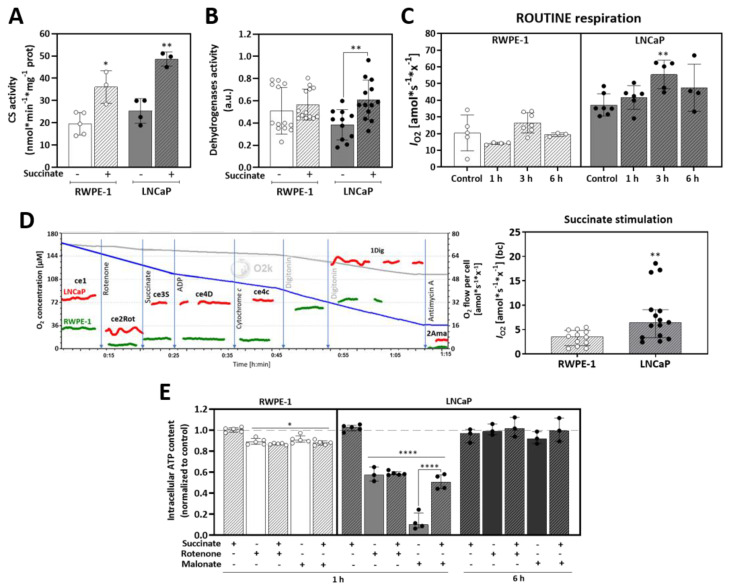
Succinate oxidation in mitochondria. (**A**) Citrate synthase activity (nmol * min^−1^ * mg^−1^ protein) with or without prior incubation with 5 mM succinate for 6 h (*N* ≥ 3). (**B**) Dehydrogenases activity measured after incubation with 5 mM succinate for 6 h using a WST-1 assay (*N* ≥ 11). (**C**) Time-course of ROUTINE respiration of living cells after incubation with 5 mM succinate for 1, 3 or 6 h (*N ≥* 4). Measurements were performed with living cells in their respective medium without supplements. (**D**) O_2_ consumption stimulated by addition of succinate (10 mM) after rotenone titration (*N* ≥ 12). Representative traces show the titration steps of the SUIT protocol: ce1, living cells (ROUTINE respiration); ce2Rot, rotenone (0.5 µM Rot for inhibition of CI, measurement of residual O_2_ consumption); ce3S, succinate (10 mM S; respiration supported by external succinate plasma membrane transport in living cells); ce4D, ADP (1 mM ADP) and ce4c, cytochrome *c* (10 µM *c* for testing the cell viability of living cells and plasma membrane integrity); 1Dig, digitonin (5 µg/mL Dig; plasma membrane permeabilization enabling the entrance of substrates for stimulation in O_2_ consumption - OXPHOS); and 2Ama, antimycin A (2.5 µM Ama for inhibition of CIII). All experiments were performed at 37 °C with a cell concentration of 1.0 × 10^6^ x/mL suspended in their respective cell culture media without supplements in the 2 mL O2k-chamber. Units are O_2_ flow per cell counts, *I*_O2_ [amol*s^−1^*x^−1^], where “x” represents the elementary unit of cell count [29], baseline corrected (bc). (**E**) ATP content after incubation of cells for 1 or 6 h with (+) or without (−) 5 mM succinate, 0.5 µM rotenone, 5 mM malonate (*N* ≥ 3). Median and IQR. Unpaired *t*-tests for independent experiments comparing +/− succinate in each condition (**A**–**D**) or ordinary one-way ANOVA tests (**E**). * *p* = 0.05; ** *p* < 0.001; **** *p* < 0.00001.

As a second effect, in addition to anaplerosis, mitochondrial respiration is expected to increase upon succinate uptake. First, to test whether succinate increases mitochondria content, citrate synthase (CS) activity was checked as a mitochondrial marker enzyme. In both cells, prior succinate incubation for 6 h almost doubled CS activity (Figure 3A) and stimulated overall activity of dehydrogenases (Figure 3B) suggesting that succinate incubation stimulates mitochondrial biogenesis and/or activity.

Next, the succinate effect on mitochondrial respiration was studied using high-resolution respirometry (O2k, Oroboros Instruments). Prior to measurements, cells were incubated with succinate for 1, 3 or 6 h and then ROUTINE respiration was measured in supplement-free basal medium. In the malignant LNCaP cells ROUTINE respiration increased with succinate incubation, with a peak after 3 h, whereas no significant changes were observed in the non-malignant RWPE-1 cells (Figure 3C). Of note, ROUTINE respiration in unstimulated LNCaP cells was about twice that of RWPE-1 cells. To confirm that the stimulation of respiration was due to succinate import and not to plasma membrane damage and consequent direct exposure of mitochondria to succinate, experiments were performed with cell viability always higher than 90 %. The cytochrome *c* flux control efficiencies and the ADP flux control efficiencies confirmed the intactness of plasma and mitochondrial membranes, respectively (Figure S3A,B).

To further investigate the succinate effect on respiration, a substrate-uncoupler-inhibitor titration (SUIT) protocol [28] was applied with cells suspended in their correspondent supplement-free culture medium. After stabilization of ROUTINE respiration, rotenone (Rot) was added to inhibit CI, blocking NADH oxidation and impairing the TCA cycle, allowing the measurement of succinate-linked respiration (S-pathway) with succinate as the only external respiratory substrate. ADP and cytochrome *c* (cyt *c*) were added to check the intactness of the plasma and outer mitochondrial membranes, respectively (Figure S3). Then, the cells were permeabilized to achieve the reference state of respiration stimulated by succinate and kinetically saturating ADP. Finally, antimycin A (Ama) was added to block the transfer of electrons to oxidized Q and inhibiting the ETS. LNCaP cells showed 2 times higher O_2_ consumption stimulated by succinate upon CI inhibition than RWPE-1 cells (Figure 3D). However, succinate stimulation did not have any effect on total cellular ATP content compared to controls after 1 h of incubation in both cell lines (Figure 3E). In RWPE-1 CI or CII inhibition with Rot or malonate (Mna), respectively, promoted a slight but significant decrease of ATP levels of about 12% in all conditions independent of succinate stimulation.

In LNCaP cells, ATP levels were decreased by 43% after CI inhibition independent of succinate stimulation. On the contrary, upon CII inhibition ATP levels decreased by 90%, and were rescued up to 50% by succinate addition. All these changes in ATP content disappeared after 6 h succinate treatment in LNCaP cells, indicating metabolic plasticity in response to disturbances. Taken together, these findings confirm a pronounced adaptative metabolic phenotype in the malignant cells with increased respiration but with no alteration of the ATP levels. On the other hand, non-malignant cells showed little effects, except a small increase of O_2_ consumption.

### 3.4. Malignant Prostate Cancer Cells Display Higher Succinate Affinity, and Succinate Incubation Induces Expression and Activity of Mitochondrial Complexes and Respiration

**Figure 4:**
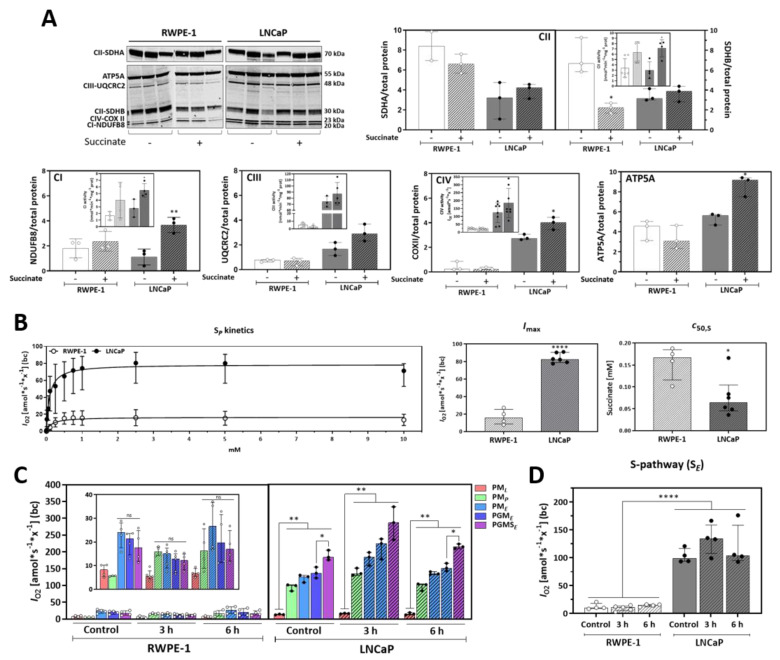
Effect of succinate on mitochondrial function. (**A**) Protein expression of subunits of electron transfer system (ETS) complexes NDUFB8 (CI), SDHA and SDHB (CII), UQCRC2 (CIII), COX II (CIV) and ATP5A (ATP synthase) (*N* ≥ 3; *n* = 2). Cells were incubated for 6 h with 5 mM succinate (+) or vehicle (-) before harvest. Proteins were normalized by total protein content within each protein extract of RWPE-1 and LNCaP cells. Inserts represent CI, CII, CIII (nmol * min^−1^ * mg^−1^ protein) and CIV activities per cell [amol*s^−1^*x^−1^] with or without 5 mM succinate incubation for 6h. *N* ≥ 3. A representative western blot image is shown on top. (**B**) Hyperbolic kinetics of succinate-linked OXPHOS capacity (S*_P_*) in MiR05 buffer. After stabilization of ROUTINE respiration, the following chemicals were added: digitonin (5 µg/mL), rotenone (0.5 µM), ADP (1 mM), and succinate in the range of 10 µM–10 mM. *I*_max_ represents maximum O_2_ flow at kinetic saturation by succinate (*N* ≥ 4) and *c*_50,S_ is the concentration of succinate at which 50% of *I*_max_ is obtained (*N* ≥ 4). (**C**) HRR analysis employing SUIT-001 O2 ce-pce D003 in RWPE-1 and LNCaP cells incubated with 5 mM succinate for 3 or 6 h. PM*_L_*, LEAK-compensatory respiration (*L*) supported by pyruvate (P) and malate (M); PM*_P_*, pyruvate & malate supported OXPHOS capacity (*P*, after addition of ADP); PM*_E_*, pyruvate & malate supported ET capacity (*E*, after uncoupler titration); PGM*_E_*, capacity supported by pyruvate, malate & glutamate (G); PGMS*_E_* ET capacity supported by pyruvate, malate, glutamate & succinate (S) (*N* ≥ 4). Insert represents a zoom in of the RWPE-1 data. (**D**) Succinate (S-) pathway respiration after addition of PGMS and subsequent inhibition of CI with Rot (S*_E_*) in cells incubated with 5 mM succinate for 3 or 6 h. All measurements at 37 °C in MiR05 with 1.0 × 10^6^ x/mL of RWPE-1 or LNCaP cells in the O2k-chamber. Units are O_2_ flow per cell counts, *I_O2_* [amol*s^−1^*x^−^^1^], where “x” represents the elementary unit of cell count [29], baseline-corrected (bc). Median and IQR. Two-tailed unpaired *t*-tests between control and each time-point or between cell lines (**A**,**B**) and two-way ANOVA for multiple comparisons (**C**,**D**). * *p* = 0.05, ** *p* < 0.001, **** *p* < 0.00001.

Increased mitochondrial oxygen consumption after incubation with succinate can be related to upregulated mitochondrial subunit content, activation of mitochondrial respiratory complexes or increased succinate affinity. Therefore, first the protein expression of the respiratory complexes’ subunits and their corresponding activities were analyzed (Figure 4A). After exposure to extracellular succinate for 6 h, increased levels of CI (NDUFB8), CIV (COXII) and ATP synthase (ATP5A) subunit proteins were detected whereas CII (SDHA and SDHB) proteins remained unchanged in LNCaP cells. Conversely, no changes in expression or activities of all complex subunits, except SDHA, were found in RWPE-1 cells upon exposure to succinate. In LNCaP cells the activities of CI and CII were stimulated by succinate incubation, but not the activities of CIII and CIV. Of note, the activities of CIII and CIV were lower in RWPE-1 in comparison to LNCaP cells, which is in line with the higher respiratory capacity of the malignant cells. 

Next, the affinity of mitochondrial respiration for succinate was investigated in permeabilized cells evaluating concentration-dependent succinate stimulation of OXPHOS capacity [39]. Cells were suspended in mitochondrial respiration medium and permeabilized with digitonin (Dig). To measure solely succinate-linked respiration, CI was inhibited, a saturating concentration of ADP was added, and succinate was titrated in steps up to 10 mM. Kinetic analyzes revealed a maximum O_2_ flow, *I*_max_, of 82.7 amol*s^−1^*x^−1^; (IQR 11.04) for LNCaP, and 15.9 amol*s^−1^*x^−1^; where “x” represents the elementary unit of cell count [29], (IQR 13.90) for RWPE-1 cells (Figure 4B). Succinate concentrations for half maximal capacity, *c*_50,S_, were 0.064 mM (IQR 0.03516) for LNCaP, and 0.167 mM (IQR 0.051) for RWPE-1 cells. These data suggest a significantly higher mitochondrial affinity for succinate in LNCaP cells. 

To further dissect the effects of succinate on mitochondria a detailed analysis of mitochondrial respiration was performed by determining the respiratory capacities triggered by different substrates fueling electrons into the ETS either through CI (pyruvate, malate, glutamate; N-pathway) or CII (succinate; S-pathway). O_2_ consumption was measured in permeabilized cells during a sequential addition of substrates following the SUIT reference protocol RP1 [28] and the contribution of each substrate to mitochondrial respiratory capacity was calculated for untreated RWPE-1 and LNCaP cells and for cells pretreated with 5 mM succinate for 3 or 6 h (Figure 4C). In RWPE-1 cells pyruvate (P) and malate (M) triggered maximal respiration in the noncoupled electron transfer state (ET pathway) and further addition of glutamate (G) and succinate (S) did not change O_2_ consumption, independent of prior incubation with succinate. Conversely, in LNCaP cells all three NADH-linked substrates (PGM) were not sufficient to achieve maximum O_2_ consumption, which was reached only after the S-pathway substrate succinate was added. Thus, in contrast to RWPE-1 cells, succinate elicited a significant additional increase in respiratory capacity in LNCaP cells. Pre-incubation of LNCaP cells with succinate for 3 or 6 h did not significantly change respiratory capacities (Figure S4). The S-pathway capacity, which was determined after Rot addition, was significantly increased in LNCaP compared to RWPE-1 cells (Figure 4D).

It has been reported that changes in extracellular pH caused by efflux of lactate as a result of increased glycolytic flux in cancer cells, can influence succinate uptake of cells expressing the NaDC3 transporter [26]. In our hands, prior incubation or incubation during the measurement in acidic pH did not influence the succinate-linked respiration in RWPE-1 nor in LNCaP cells (Figure S5).

Together, these results show that mitochondria of the malignant prostate cells have a higher affinity for succinate and this metabolite supports mitochondrial physiology through the induction of expression and activities of mitochondrial complexes. Moreover, LNCaP cells rely on succinate-linked respiration to reach maximum mitochondrial respiratory capacity.

### 3.5. External Succinate Stimulates Migration, Invasion, and Colony Formation, but Not Proliferation, Particularly in Malignant Prostate Cells

**Figure 5:**
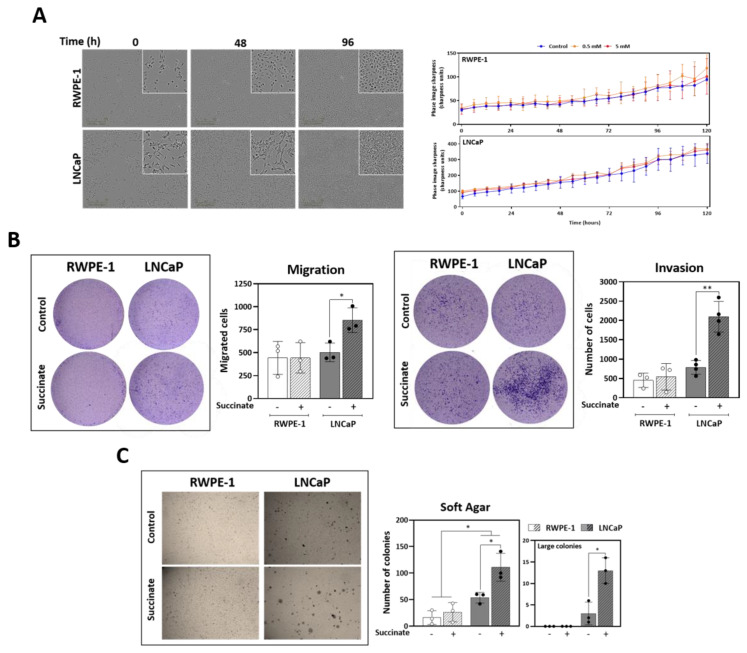
Proliferation, motility and colony formation. (**A**) Proliferation assay over the course of 120 h with cells incubated with 0.5 or 5 mM succinate or vehicle (*N* ≥ 4, *n* = 2). (**B**) Transwell cell migration and invasion capacity in Matrigel™, respectively, during 48 h incubation with 5 mM succinate or vehicle (*N* ≥ 4, *n* = 3). (**C**) Anchorage-independent growth colony formation in soft agar. Cells were incubated with 5 mM succinate or vehicle for 2 weeks (*N* ≥ 4, *n* = 2). Median and IQR. Paired *t*-tests and Wilcoxon matched-pairs signed rank between control and treated cells. * *p* = 0.05, ** *p* < 0.001.

Following the observations on the effect of succinate on cell metabolism, the contribution of external succinate to key properties of tumor progression and metastasis, such as proliferation and tumorigenesis through invasion and migration was investigated. Interestingly, incubation of cells with 5 mM succinate for up to 120 h did not stimulate proliferation in prostate cells (Figure 5A). However, succinate increased migration and invasion of LNCaP but not of RWPE-1 cells (Figure 5B). The formation of colonies independent of anchorage in soft agar was also checked. Succinate stimulated formation of colonies in both cell lines, however LNCaP cells presented more, and larger colonies compared to the controls (Figure 5C). Altogether, these data suggest that succinate increases the tumorigenic potential in LNCaP cells, but not proliferation.

### 3.6. Anaplerotic and Bioenergetic Effects of Succinate Are Linked to Prostate Cancer Poor Prognosis

**Figure 6:**
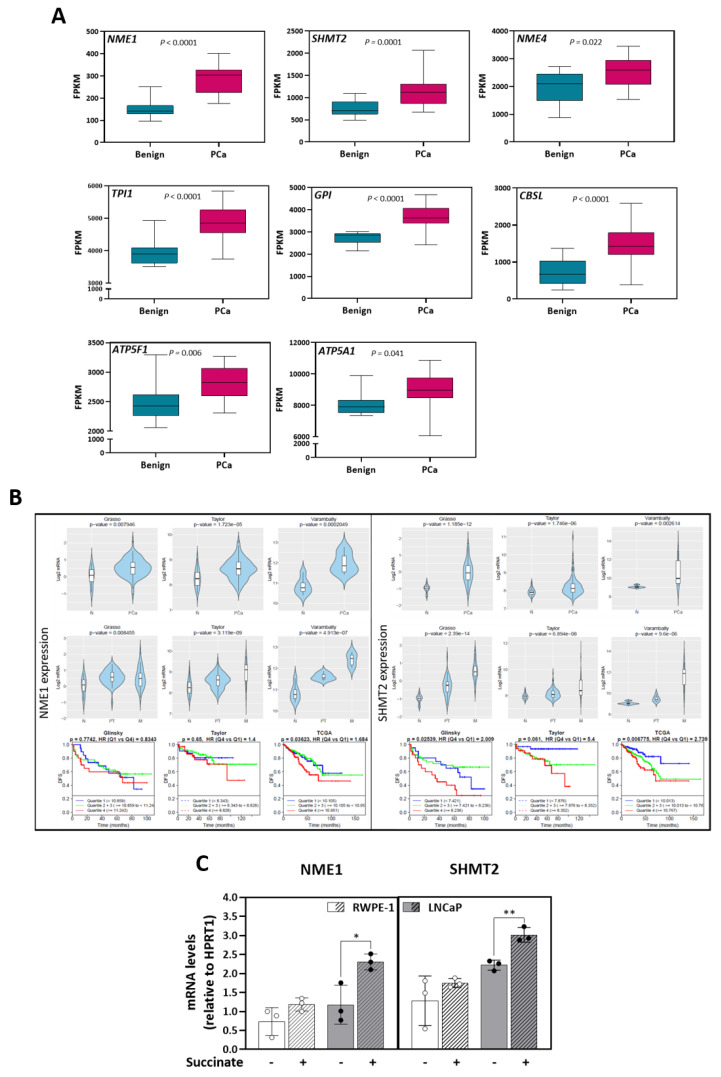
Expression of enzymes linked to metabolites stimulated by succinate in transcriptomic cohorts of prostate cancer patients. (**A**) Boxplots representing Fragments Per Kilobase Million (FPKM) values for expression of significantly upregulated genes in prostate cancer (PCa) (pink) vs. the paired benign (blue) biopsies (*N* = 16, [25]). Data are presented as boxplots with median, 25^th^–75^th^ percent percentile (box). Multiple *t*-test followed by Benjamini–Hochberg correction for multiple testing. (**B**) Significantly increased mRNAs were analyzed using Cancer Tool free software [31] in different prostate cancer transcriptome datasets. The violin plots show the expression of NME1 and SHMT2 comparing normal tissue (N) and prostate cancer (PCa, upper panel), and metastasis when given (primary tumor, PT; metastasis M, middle panel). The *Y*-axis represents log2-normalized gene expression. Statistical significance between N and PCa was tested using t-test and between N, PT and M using Anova. For disease-free survival (DFS) analysis, Kaplan-Meier curves were divided into 4 quartiles according to gene expression: Q1 (Blue), Q2 + Q3 (Green), Q4 (Red) (bottom panel). Each curve represents the percentage of the population that exhibits recurrence of the disease in function of time. A Mantel-Cox test was performed to compare the differences between curves, while a Cox proportional hazards regression model was performed to calculate the Hazard Ratios (HR). Datasets: Glinsky et al. [33] (*N* = 79); Grasso et al. [34] (*N* = 88); Taylor et al. [35] (*N* = 179); Varambally et al. [36] (*N* = 19); TCGA-PRAD (*N* = 496). (**C**) NME1 and SHMT2 mRNA expression with or without succinate (5 mM) for 48 h in RWPE-1 and LNCaP cells. mRNA levels are expressed normalized by the endogenous control HPRT1 (*N* = 3; *n* = 2). Median and IQR. Two-tailed unpaired *t*-tests between control (−) and succinate (+). * *p* = 0.05, ** *p* < 0.001.

In an effort to link the metabolic alterations and the increase in tumor potential detected in our prostate cell models to human disease, expression of enzymes associated with the metabolites specifically increased in LNCaP cells upon succinate stimulation was checked in the transcriptome dataset of 16 paired cancer (PCa) and benign (BE) samples previously published by our group [25] (Table 1). Eight of the 26 genes (30.8%) linked to elevated metabolites were found significantly overexpressed in tumor compared to benign tissue: NME/NM23 nucleoside diphosphate kinase 1 and 4 (NME1 and 4) involved in mitochondrial dynamics and turnover, triosephosphate isomerase 1 (TPI1) catalyzing the isomerization of glyceraldehyde 3-phosphate, glucose-6-phosphate isomerase (GPI) involved in the interconversion of glucose-6-phosphate and fructose-6-phosphate, serine hydroxymethyltransferase 2 (SHMT2) catalyzing the reversible reaction of serine and tetrahydrofolate to glycine and 5,10-methylene tetrahydrofolate, cystathionine beta-synthase like (CBSL) involved in the elimination of L-methionine; ATP synthase F1 subunit alpha (ATP5F1) and ATP synthase peripheral stalk-membrane subunit B (ATP5F1), two subunits of the ATP synthase complex (Figure 6A). 

Further examination in publicly available prostate cancer transcriptome datasets [32,33,34,35] confirmed NME1 and SHMT2 as consistently upregulated in prostate tumor cohorts (Figure 6B). Tumors with high expression of NME1 displayed a significantly shorter progression free survival in the TCGA dataset (comparison of highest to lowest expression quartiles), and SHMT2 in the TCGA and in the Glinsky dataset (Figure 6B). In line with these results, stimulation of RWPE1 and LNCaP cells with 5 mM of succinate for 48 h enhanced the mRNA of both genes in the malignant cells (Figure 6C). These results indicated a link between succinate stimulation, higher malignant potential, and poor outcome in prostate cancer.

## 4. Discussion

In cancer cells, succinate plays a role as mitochondrial fuel substrate linking the TCA cycle to the electron transfer system (ETS). To meet these demands, succinate is mainly produced endogenously by succinyl coenzyme A synthetase from succinyl-CoA or it can be transported from the extracellular environment through the plasma membrane via dicarboxylic transporters [38] in some cell types. Prostate cancer cells are known to display enhanced mitochondrial respiration compared to healthy prostate [26,37,40]. Succinate accumulation in prostate tumor cells was found associated to loss of the tumor suppressor phosphatase and tensin-homolog deleted from chromosome ten (PTEN) [37]. PTEN acts as a tumor suppressor, decreasing phosphatidylinositol-3-kinase (PI3K) signaling. Its loss is frequently found in prostate tumors, particularly in advanced, metastatic tumor stages [41]. Zhunussova et al. stated that glucose is not the main substrate for energy supply in prostate cancer, which conversely utilize metabolites from the microenvironment, such as succinate, to support bioenergetics [26]. Moreover, some cell lines express the dicarboxylic transporters NaDC3 and/or NaCT [26,37], which can be directly linked to succinate uptake. In the present work, the transport of exogenous succinate and its endogenous fate mediating bioenergetic metabolism and tumorigenesis were verified. 

The focus of this study is a comparison of cells representing an early tumor stage with a high risk of progression, LNCaP cells, and nonmalignant prostate epithelial cells, derived from normal prostate tissue, RWPE-1 cells. LNCaP cells are highly androgen-responsive, PTEN negative and are prone to progress to castration-resistance upon androgen deprivation therapy, thus mimicking the natural course of initial progression of this disease [42]. This probably represents the most crucial, irreversible step of prostate cancer progression to a deadly disease and pro-oncogenic effects at this point are of clinically relevance.

First, expression of the succinate transporters was confirmed in the non-malignant RWPE-1 and malignant LNCaP prostate cell models (Figure 1A,B). Moreover, experiments using labelled succinate showed that both cells take up succinate efficiently (Figure 1C). Fingerprint non-targeted metabolomics demonstrated intracellular succinate accumulation over time when cells were incubated with succinate (Figure 1D). Of note, whilst the uptake rate peaked at 3 h and was low after 6 h of incubation in RWPE-1 cells succinate further accumulated up to 24 h, peaking at 12 h with concentrations in the millimolar range (approx. 2 mM) in LNCaP cells. 

A potential limitation of our study concerns the distinct tissue/patient origins of the prostate cell lines and their different cell culture medium in vitro. The literature points out the impact of cell culture media in metabolomic profiles [43,44]. Therefore, both cell lines were kept in their specific medium to avoid bias and metabolite values were normalized to each cell lines’ mock treatment control samples. Furthermore, high-resolution respirometry measurements were performed in the respective basal media without supplements (FBS or BPE, respectively). Both media contain very similar concentrations of glucose, glutamine, and amino acids and do not contain succinate or citrate.

To investigate the fate of extracellular succinate in the prostate cells, metabolomics analyzes were performed (Figure 2). TCA cycle intermediaries were increased in both cell types, earlier in LNCaP than in RWPE-1 cells except αKG, which was decreased. This result suggests that extracellular succinate has an anaplerotic effect and induces a potential efflux of the αKG to other pathways, most likely conversion into glutamate. The TCA cycle is the central hub of carbon metabolism that fuels biosynthetic precursors for building blocks. Anaplerosis is the property of replenishing the TCA cycle. Glutamine, the most abundant amino acid in mammals [45], is the most important anaplerotic compound. Glutamine is converted into glutamate through glutaminases (GLS1 and GLS2) and then further to αKG by glutamate dehydrogenase (GLUD1 or GLUD2) or it can be excreted [45]. αKG can also be produced by oxidative decarboxylation of isocitrate, catalyzed by isocitrate dehydrogenases (IDH1, IDH2 or IDH3) [46]. Moreover, biosynthesis of amino acids depends on the availability of αKG and glutamate for transamination reactions. Other tricarboxylic acids such as malate and pyruvate also display may replenish the TCA. Additional experiments using labeled succinate would enable the analyzes of the metabolic fate of the carbons of succinate. 

In order to consider the biological pathways that may underlie the alterations in metabolite levels, attention was focused on those enzymes known to catalyze production of the metabolites in question [47] (Table S2). Based on this approach, energy and amino acid metabolism, proliferation, fatty acid metabolism, mobility and cell adhesion, cell death, and chronic diseases were identified as associated pathways (Figure S2).

The literature shows the relevance of succinate in mitochondrial physiology and homeostasis [48]. Both cell types increased TCA cycle metabolites. In general, response to exogenous succinate supply was faster and differences achieved higher significance in the malignant LNCaP compared to the non-malignant RWPE-1 cells (Figure 2). Moreover, TCA metabolites decreased in LNCaP cells beyond 6 h of succinate supply. These findings suggest that RWPE-1 cells have a more robust and buffered metabolism, whilst LNCaP cells have more plasticity that results in faster rearrangement of metabolism in response to externally supplied substrates compared to RWPE-1. 

Furthermore, intramitochondrial utilization of succinate in supporting mitochondrial function was investigated by analyzing expression of mitochondrial proteins and the activities of complexes, and by high-resolution respirometry. Succinate incubation increased citrate synthase activity in both cell lines and, in LNCaP cells, increased dehydrogenases activity (Figure 3A,B), indicating stimulation of mitochondrial function, or possibly an increase in mitochondrial mass. Additionally, a significant increase of ROUTINE respiration was observed after incubation with succinate in LNCaP cells (Figure 3C). As described previously in malignant prostate tissue biopsies [25], succinate can compensate for CI mutations and recover full respiratory capacity, supplying electrons to the ETS through CII. In view of that, a SUIT protocol was applied for analysis of succinate stimulation after CI inhibition (Figure 3D). These findings suggest that succinate stimulates mitochondrial function primarily in the malignant prostate cells.

Short term succinate treatment did not alter the ATP content in prostate cells and did not rescue the ATP decline induced by rotenone or the competitive inhibitor malonate, except for malonate in LNCaP cells (Figure 3E). When cells were incubated for 6 h, no effect of the inhibitors was observed showing that it is a transient effect and that the cells are capable to recover steady-state levels of ATP rapidly. Our results show that in malignant prostate cells, succinate increases respiration without changing ATP levels. In contrast, RWPE-1 cells seem to have compensatory mechanisms that rapidly limit the impact of external succinate on overall mitochondrial function. 

Mitochondrial utilization of succinate increased the proteins and activities of ETS complexes in LNCaP cells (Figure 4A). CII activity was increased despite unchanged SDHA and SDHB protein levels, supporting a succinate-dependent mitochondrial regulatory mechanism. Also, CI and CII activities were stimulated significantly in LNCaP but not CIII and CIV activities. CIII receives the electrons from Q and transfers to CIV, which consumes O_2_ and reoxidizes cyt *c*. In LNCaP cells, both expression and activity of CIII and CIV were high in comparison to RWPE-1, supporting the fact that LNCaP cells respire more. Moreover, a much lower affinity for succinate of RWPE-1 compared to LNCAP cells was observed. Succinate-linked respiratory capacity in the intracellular concentration range (up to 100 µM [48]) was significantly increased in the malignant cells (Figure 4B).

To understand the flux of substrates through the TCA cycle and their anaplerotic capacity, respiration in permeabilized cells was triggered by addition of NADH-linked substrates and succinate. In RWPE-1, pyruvate (P) and malate (M) stimulated the maximum respiratory capacity when the respiration is noncoupled, turning on the ET-pathway (E). Glutamate (G) and succinate (S) did not further increment the oxygen consumption. Conversely, respiration in LNCaP revealed that succinate is required to achieve the maximum oxygen consumption capacity besides P, M and G additions (Figure 4C).

For several types of cancer, it has been shown that anaplerosis, mainly supported by glutamine, plays a critical role in proliferation and tumorigenicity [49,50]. Succinate stimulates amino acids metabolism, mainly through the accumulation of methionine, argininosuccinate, and aspartate and this phenotype has been described to impact tumorigenesis and increase malignancy [51,52]. Succinate did not stimulate proliferation, but it promoted tumorigenic potential through migration, invasion, and anchorage-independent growth. This indicates a succinate oncogenic-driven potential in prostate cancer (Figure 5A–C).

To link our findings in cell lines to human prostate tumors, a transcriptomic data set of paired non-malignant/malignant prostate tissue samples previously generated by our group [25] was consulted to examine differential expression of genes encoding enzymes associated with metabolite alterations (Table S5). Genes associated to significantly accumulated metabolites in LNCaP cells were cross-validated in other publicly available gene expression datasets. *NME1* and *SHMT2* genes are more expressed in cancer tissues and in metastatic samples when compared to normal tissues in several datasets. Importantly, high expression of *NME1* was associated to a worse disease-free survival in prostate cancer patients in the TCGA dataset, and high *SHMT2* expression in the TCGA and the Glinsky datasets (Figure 6B). In line with these data, mRNA expression analyzes showed that the succinate stimulates *NME1* and *SHMT2* expression in LNCaP cells (Figure 6C).

NME1 and other members of the NME family have nucleoside diphosphate kinase activity (NDPK), playing a major role in the synthesis of nucleoside triphosphates other than ATP [53]. Some studies show that NDPKs can be localized in the mitochondrial matrix, in the vicinity of succinyl-CoA synthetase, which is the only enzyme in the TCA cycle that uses GTP/GDP as a cofactor for production of succinate from succinyl-CoA or vice versa [54,55]. Moreover, NME1 has been shown to regulate global gene expression profiles in breast carcinoma cell lines with the lysophosphatidic acid receptor EDG2 identified as a motility-driving target of NME1-mediated tumor suppression [56]. Reduced expression has been observed in metastases and advanced forms of thyroid carcinoma suggesting a tumor suppressor activity [57,58]. However, in other tumors, upregulated NME1 levels have been correlated with poor prognosis, especially in neuroblastoma [59] and some forms of leukemia and lymphoma [60,61]. NME1 is also expressed in some metastatic prostate cell lines, and it has been proposed as a prognostic tissue marker [62].

Serine hydroxymethyltransferases (SHMT) 1 (cytoplasmic) and 2 (mitochondrial) are responsible for serine metabolism to produce glycine essential for GSH, heme synthesis, and 5,10-methylentetrahydrofolate, a one-carbon unit carrier indispensable for several anabolic pathways, including de novo nucleotide biosynthesis [63,64]. This enzyme reaction can also run in the opposite direction, producing serine from glycine at the cost of one-carbon units [65,66]. Recent studies have shown that cancer cells require SHMT (particularly SHMT2) for optimal proliferation and tumorigenicity, indicating the importance of serine catabolism in cancer [67,68]. The direction of serine/glycine conversion is also an important factor in cancer cell metabolism, and it has been suggested that SHMT2 is the major serine-to-glycine conversion enzyme supporting tumorigenesis [69]. Recently, a potential role of SHMT2 has been proposed as a modulator of STAT3, a master regulator of energy metabolism, which is involved in the transition of prostate cancer towards a more aggressive phenotype [70].

## 5. Conclusions

The present study unravels the fate of exogenous succinate in malignant and non-malignant prostate cancer cells. Our data confirm an onco-driving potential of succinate contributing to the malignant phenotype and tumorigenesis by enhancing mitochondrial function through support in anaplerosis and as a substrate fueling energy metabolism. To our knowledge, this is the first time that anaplerosis of succinate is reported. Also, expression of *NME1* and *SHMT2* is linked to poor prognosis in cancer patients, and in prostate cancer cells succinate enhances their expression.

## Figures and Tables

**Table 1:** Genes encoding enzymes related to metabolites specifically increased in malignant LNCaP. -Eight genes displayed increased expression in PCa compared to paired benign tissue samples in the cohort of 16 prostate cancer patients [25], *NME1, GPI, TPI1, CBSL, SHMT2, ATP5F1, NME4* and *ATP5A1*. *NME1* and *SHMT2* genes (in bold) were selected as markers of poor prognosis based on confirmation of overexpression in tumors in additional publicly available datasets and association with shorter disease-free survival (Figure 6).

Associated with Increased Metabolites in Malignant LNCaP Cells				
Genes	Mean_Benign	Mean_PCa	*p* value_PCa_VS_Benign	*p* adj_PCa_VS_Benign
***NME1***	**149.36**	**284.76**	**8.050 × 10^−15^**	**2.969 × 10^−12^**
*GPI*	2753.64	3682.55	1.523 × 10^−09^	7.252 × 10^−08^
*TPI1*	3976.64	4868.08	9.558 × 10^−08^	2.137 × 10^−06^
*CBSL*	717.07	1481.50	7.901 × 10^−06^	7.883 × 10^−05^
***SHMT2***	**753.85**	**1120.12**	**2.331 × 10^−05^**	**1.953 × 10^−04^**
*ATP5F1*	2475.32	2818.06	1.664 × 10^−03^	6.747 × 10^−03^
*SHMT1*	501.89	419.46	5.905 × 10^−03^	1.905 × 10^−02^
*NME4*	1990.21	2545.71	7.191 × 10^−03^	2.238 × 10^−02^
*ATP5A1*	8055.99	8915.23	1.514 × 10^−02^	4.106 × 10^−02^
*PGAM1*	414.10	351.19	1.586 × 10^−02^	4.264 × 10^−02^
*GLUL*	9047.37	7134.23	1.859 × 10^−02^	4.844 × 10^−02^
*CPT1C*	75.95	57.83	2.470 × 10^−02^	6.129 × 10^−02^
*PSPH*	239.95	281.43	2.833 × 10^−02^	6.809 × 10^−02^
*ABAT*	2038.80	3005.96	3.597 × 10^−02^	8.252 × 10^−02^
*G6PC*	0.16	0.81	4.683 × 10^−02^	1.022 × 10^−01^
*ALDH7A1*	2078.30	2557.72	5.704 × 10^−02^	1.197 × 10^−01^
*NME2*	15.21	22.29	1.345 × 10^−01^	2.333 × 10^−01^
*CPT1B*	3.34	4.90	1.510 × 10^−01^	2.558 × 10^−01^
*NME3*	472.52	621.70	1.655 × 10^−01^	2.745 × 10^−01^
*UPB1*	6.78	5.83	3.837 × 10^−01^	5.170 × 10^−01^
*GLS*	1539.22	1470.24	4.210 × 10^−01^	5.523 × 10^−01^
*G6PC2*	1.28	0.96	4.587 × 10^−01^	5.867 × 10^−01^
*PHGDH*	2123.91	2000.81	6.254 × 10^−01^	7.300 × 10^−01^
*PGK1*	4894.12	4979.30	6.473 × 10^−01^	7.476 × 10^−01^
*PGAM2*	7.62	7.41	6.823 × 10^−01^	7.758 × 10^−01^
*CPT1A*	1826.32	1809.73	9.266 × 10^−01^	9.522 × 10^−01^

## Data Availability

For in-silico expression analysis the following publicly available prostate cancer transcriptome data sets were used: Schöpf et al. [25]; Glinsky et al. [32]; Grasso et al. [33]; Taylor et al. [34]; Varambally et al. [35]; TCGA-PRAD (https://cancergenome.nih.gov/ Accessed on 1 February 2021).

## Supplementary Materials

The following data are available online at https://www.mdpi.com/article/10.3390/cancers13071727/s1, Figure S1: Representative trace of succinate dose-effect on respiratory capacity, Figure S2: Metabolomics computational analysis, Figure S3: Viability controls, Figure S4: Respiratory capacities, Figure S5: Succinate-linked respiration at different medium pH. Figure S6: Western blot raw images. Table S1: Metabolomics normalized data, Table S2: Proteins associated to metabolites, Table S3: Description of metabolite associated enzymes, Table S4: KEGG pathway analyzes relating each metabolic pathway with the enzymes found by manual curation in the metabolomic data, Table S5: Transcriptomic data of genes encoding enzymes of interest displaying the expression in paired benign and malign prostate tissue samples.

## Abbreviations

TCA—Tricarboxylic acid; SDH—Succinate dehydrogenase; CI—Complex I; CII—Complex II; ET—Electron transfer; HIF-1α—Hypoxia inducible factor 1α; PHD—Prolyl hydroxylases; ROS—Reactive oxygen species; OXPHOS—Oxidative phosphorylation; RET—Reverse electron transfer; CS—Citrate synthase; TMPD—Tetramethyl-p-phenylenediamine; SUIT—Substrate-uncoupler-inhibitor titration; ETS—Electron transfer system; Rox—Residual oxygen consumption; FCR—Flux control ratios; IQR—Interquartile range; NaDC3—Sodium-dependent dicarboxylate transporter member 3; NaCT—Sodium-dependent citrate transporter member 5; αKG—αKetoglutarate; GSH—Glutathione; ADP—Adenosine diphosphate; ATP—Adenosine triphosphate; Rot—Rotenone; cyt *c*—Cytochrome *c*; Ama—Antimycin A; Dig—Digitonin; P—Pyruvate; M—Malate; G—Glutamate; S—Succinate; BE—Benign; PCa—Prostate cancer; TPI1—Triosephosphate isomerase 1; NME1/4—NME/NM23 nucleoside diphosphate kinase 1 and 4; GPI—Glucose-6-phosphate isomerase; SHMT2—Serine hydroxymethyltransferase 2; CBSL—Cystathionine beta-synthase like; ATP5F1—ATP synthase F1 subunit alpha; ATP5F1—ATP synthase peripheral stalk-membrane subunit B; FPKM—Fragments per kilobase million; N—Normal tissue; PT—Primary tumor; M—Metastasis; DFS—Disease-free survival; PTEN—Phosphatase and tensin-homolog deleted from chromosome ten; PI3K—Phosphatidylinositol-3-kinase; GLS—Glutaminases; IDH—Isocitrate dehydrogenases; NDPK—Nucleoside diphosphate kinase activity.
