# Supplementary material for: Succinate Anaplerosis Has an Onco-Driving Potential in Prostate Cancer Cells
Source: Cancers (Basel). 2021 Apr 6;13(7):1727. doi: 10.3390/cancers13071727 (PMC8038717; doi:10.3390/cancers13071727)

Succinate Anaplerosis has an Onco-Driving Potential in Prostate Cancer Cells

Ana Carolina B. Sant’Anna-Silva, Juan A. Perez-Valencia, Marco Sciacovelli, Claude Lalou, Saharnaz Sarlak, Laura Tronci, Efterpi Nikitopoulou, Andras T. Meszaros, Christian Frezza, Rodrigue Rossignol, Erich Gnaiger and Helmut Klocker

Methods

Metabolomics computational analysis

After acquisition and identification of all metabolites present in the samples, we normalized the results for each time point of succinate incubation (1, 3, 6, 12 or 24 h) and its control (vehicle) for each cell line separately. Then, we performed statistical analysis and clustered the metabolites into groups classified as REDUCED, UNCHANGED and INCREASED according to significance. To correlate metabolite changes to metabolic pathways, enzymes involved in the production of the respective metabolite were identified by manual curation, which resulted in a list of 148 enzymes, with 2 or more enzymes associated with each metabolite. STRING analysis (© STRING CONSORTIUM 2020, http://string-db.org) was performed to establish functional protein association networks and identify enriched KEGG pathways in which these enzymes are involved. All pathways were categorized in biological function groups: energy metabolism; biosynthesis and metabolism of amino acids; proliferation; fatty acids metabolism; motility and adhesion; cell death; chronic diseases.


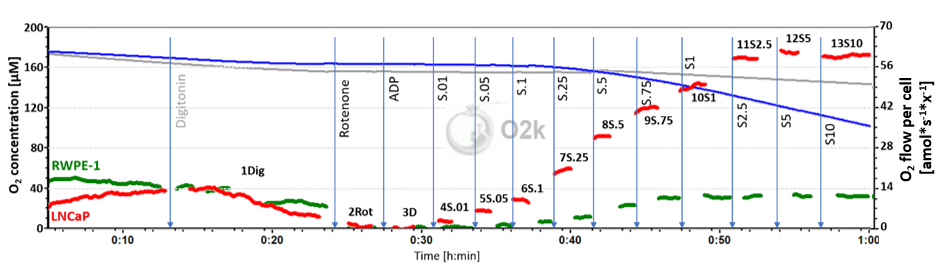


**Figure S1.** Representative trace of succinate dose-effect on respiratory capacity. O_2_ consumption was measured in MiR05 buffer using the Oroboros O2k HRR instrument. For establishing the optimal concentration of succinate yielding maximal stimulation of oxygen consumption, succinate was titrated up to a concentration of 10 mM. After stabilization of ROUTINE respiration, the following chemicals were added: digitonin (5 µg/mL, 1Dig), rotenone (0.5 µM, 2Rot), ADP (1 mM, 3D) and finally succinate in concentration steps from 0.01–10 mM (4S.01 - 13S10). Grey and blue lines represent O_2_ concentration [µM], and green and red lines represent O_2_ flow per cell [amol*s^-1^*x^-1^] for RWPE-1 and LNCaP cells, respectively.


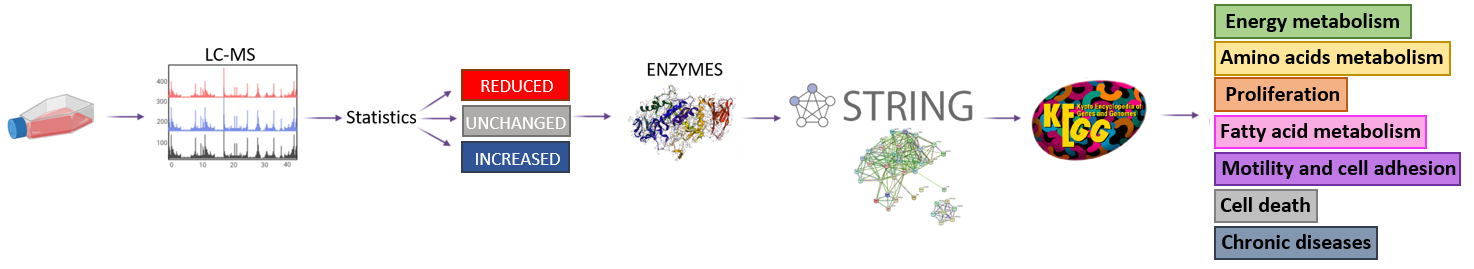


(A)


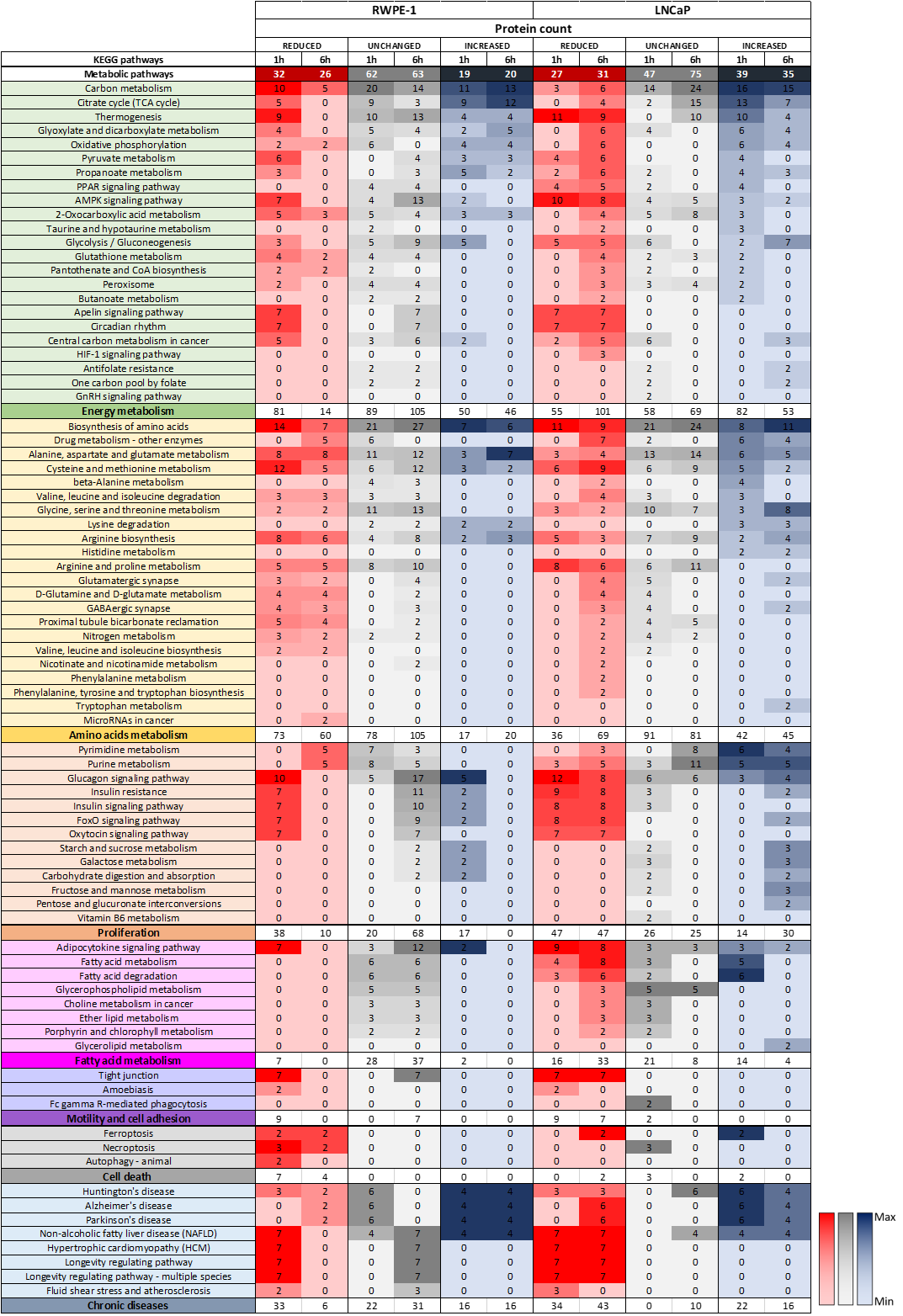


(B)

**
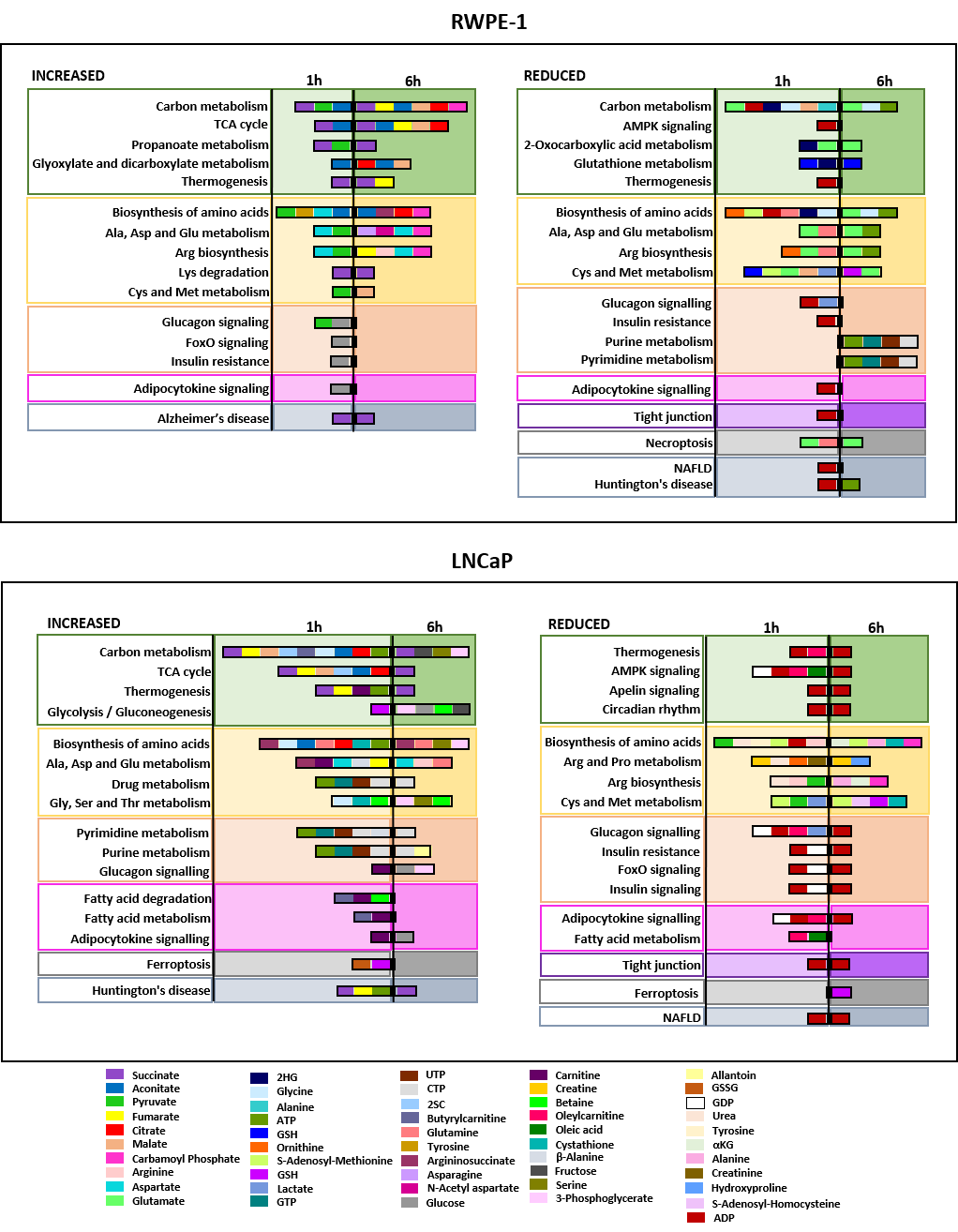
**

(C)

**Figure S2.** Metabolomics computational analysis. (**A**) Flowchart showing the design of the metabolomics computational analysis to link succinate-triggered metabolite alterations to metabolic pathways and biological functions. See Material and Methods section for detailed description of analysis. (**B**) Heatmap showing the identified pathways and the number of related proteins found, according to KEGG pathways analyzed by STRING and clustered in REDUCED, UNCHANGED and INCREASED for 1 or 6 h of succinate incubation. The darker the color, the more related proteins are assigned to that pathway. Blue color refers to REDUCED, grey to UNCHANGED, and red to INCREASED. Metabolic pathways are grouped according to the following biological function groups (left column): energy metabolism in green; biosynthesis and metabolism of amino acids in yellow; proliferation in orange; fatty acids metabolism in pink; motility and adhesion in purple; cell death in grey; chronic diseases in blue. The order of the pathways was determined following the total number of proteins per pathway in each cell line, and then sorted in decreasing order starting with LNCaP cells. (**C**) Association of metabolites changed by incubation with 5 mM of succinate for 1 or 6 h, respectively, with KEGG pathways. All associated metabolites have single color code, and the bars are divided in INCREASED and REDUCED in comparison to the controls for RWPE-1 and LNCaP cells, respectively. The pathways and the correspondent metabolites are separated into biological function groups as listed above, following the same color code.

(A)

(B)

**Figure S3.** Cell viability controls. (**A**) Cell viability measured by Trypan Blue exclusion dye (*N* = 10). (**B**) Flux control efficiency determined by cytochrome *c* addition (*j_c_*) and flux control efficiency determined by ADP addition (*j*_D_) (*N* = 4). Median and IQR.

**Figure S4.** Respiratory capacities. LEAK respiration, OXPHOS capacity and net ET capacity (PM*_E_* - PM*_L_*) from SUIT-001 experiments comparing untreated RWPE-1 and LNCaP cells with cells treated with succinate for 3 or 6 h prior to the measurements (*N* ≥ 4). Median and IQR. Two-tailed unpaired *t*-tests for comparisons to controls and ordinary one-way ANOVA for comparison of cell lines. *** *p* < 0.0001, **** *p* < 0.00001.

**
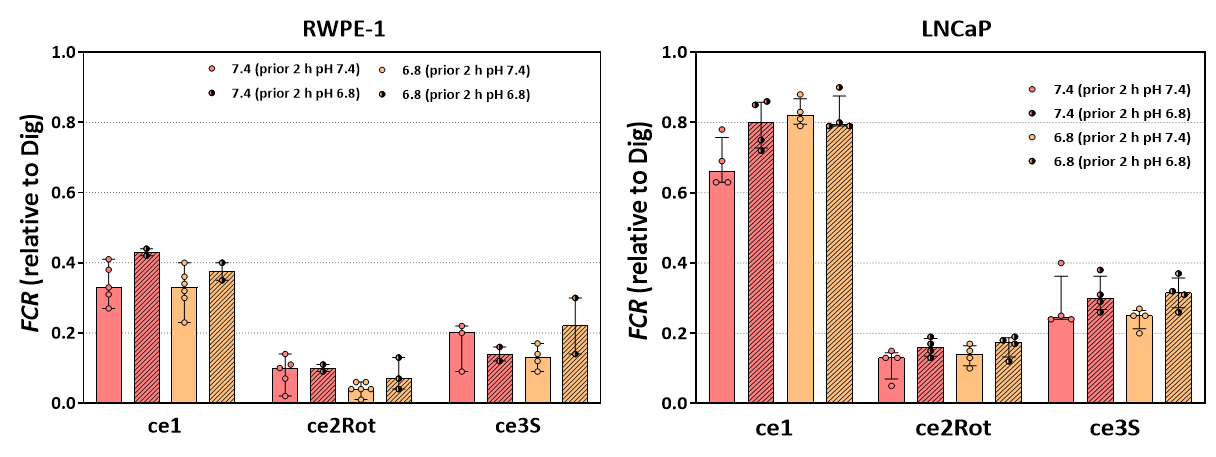
**

**Figure S5.** Succinate-linked respiration at different medium pH. Cells were incubated prior to HRR measurements for 2 h in their respective full medium adjusted to pH 7.4 or pH 6.8. Cells were then harvested and placed in the O2k chamber in their basal medium adjusted to pH 7.4 or pH 6.8 for respiratory measurements. Flux control ratios (*FCR*) relative to maximum respiration (Dig) are given for RWPE-1 and LNCaP cells. ce1, ROUTINE respiration; ce2Rot, rotenone (0.5 µM); ce3S, succinate (10 mM) (*N* ≥ 2). Median with range. Two-way ANOVA and Tukey’s multiple comparisons test.

**Figure S6 Western blot raw images**

**Figure 1A**


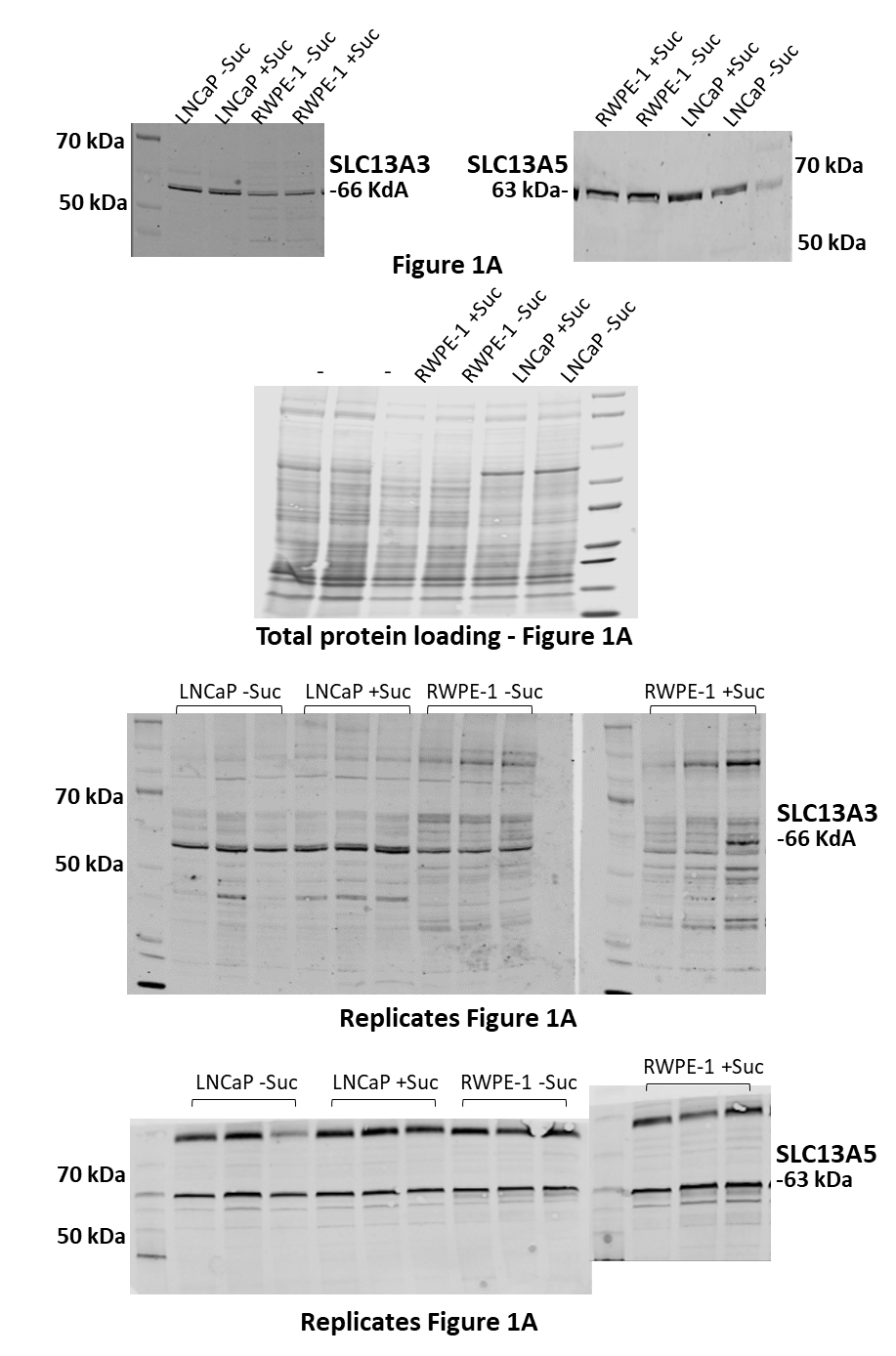


**
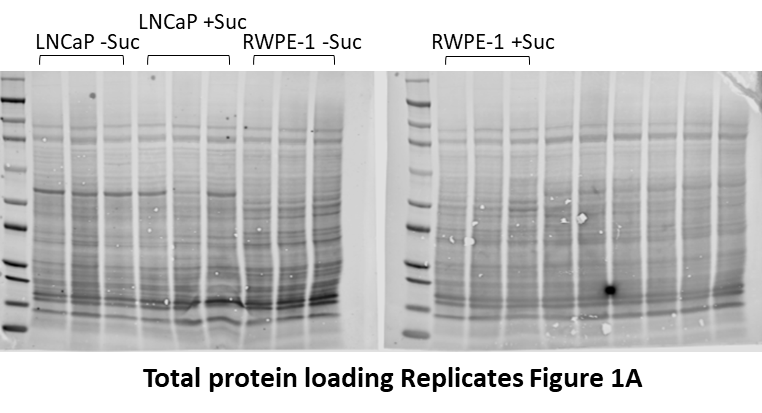
**

**Figure 4A**


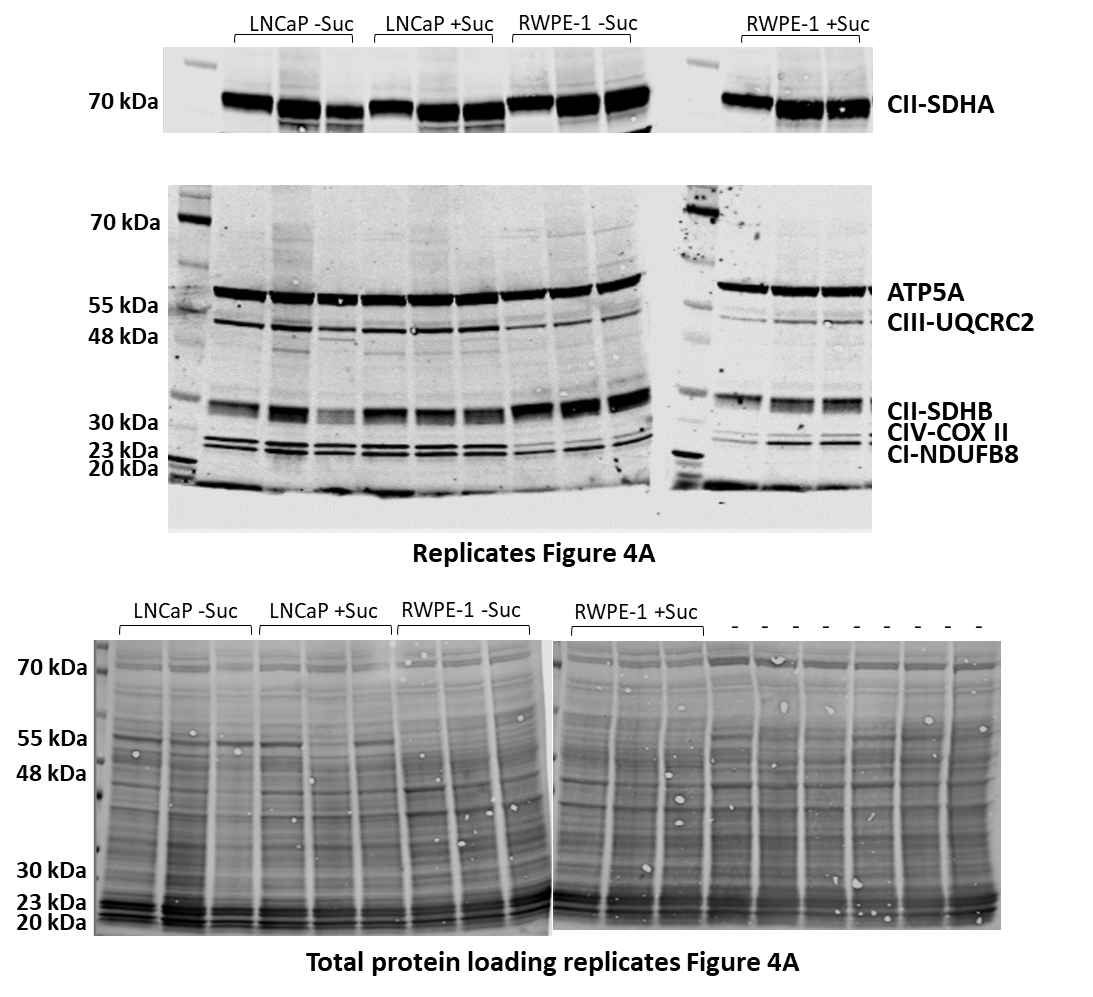

Supplement: Supplementary file 1 [file cancers-13-01727-s001.zip › cancers-1142827-supplementary-final/Supplementary Figures.docx]
